# The characteristics of stem cells in human degenerative intervertebral disc

**DOI:** 10.1097/MD.0000000000007178

**Published:** 2017-06-23

**Authors:** Lin Liang, Xuefeng Li, Dapeng Li, Weimin Jiang, Heng Wang, Jie Chen, Zhiyong Sun, Niannian Zhang, Yangyi Zhu

**Affiliations:** a Department of Orthopaedics, Shangyu People's Hospital, Shaoxing; b Department of Orthopaedics, The First Affiliated Hospital of Soochow University, Suzhou; c Department of Orthopaedics, The Affiliated Hospital of Jiangsu University, Zhenjiang, China.

**Keywords:** genes, induced differentiation, intervertebral disc degeneration, stem cells

## Abstract

**Background:**

The aim of this study is to identify which possessed the best stem-cell-like characteristics in 3 kinds of cell in human degenerative intervertebral disc: NPSCs (nucleus pulposus-derived stem cells), AFSCs (annulus fibrosus-derived stem cells), or CESCs (cartilage endplate-derived stem cells).

**Methods:**

We separated the disc samples obtained from 15 surgically treated patients with disc degenerative diseases into nucleus pulposus, annulus fibrosus, and cartilage endplate. After cultivating, we used the cell counting kit-8 to analysis the cell activity of 3 kinds of disc tissue-derived stem cell separately; different stem cells were defined with multilineage (osteogenic, chondrogenic, and adipogenic) differentiation. We extracted the total RNA and detected the expression of different lineage differentiation-related genes with the real-time polymerase chain reaction (RT-PCR).

**Results:**

Cell morphology of NPSCs, AFSCs, and CESCs did not show significant difference. Cell proliferation capacity of NPSCs and AFSCs was stronger than that of CESCs. The differentiation outcomes showed that osteocyte-like cells were stained red by Alizarin red S, chondrocyte-like cells blue by toluidine blue, and adipocyte-like red by oil red O. The RT-PCR reflected that the expression of different lineage differentiation-related genes of AFSCs was stronger than NPSCs and CESCs.

**Conclusion:**

In conclusion, we found that the cell morphology was not significantly different among NPSCs, AFSCs, and CESCs. Both differentiation and RT-PCR tests demonstrated that AFSCs had the best stem-cell-like characteristics in the human degenerative intervertebral disc.

## 1 Introduction

In the modern society, low back pain (LBP) is a very commonly seen disease that causes disability and decline of productivity, whereas degenerative disc disease (DDD) is considered to be one of the main causes of LBP.^[1]^ According to statistics, about 80% of human beings have symptoms of LBP in life and 40% suffer from the DDD.^[2,3]^

Intervertebral disc is a nonvascular structure composed of 3 special anatomical tissues, including nucleus pulposus, annulus fibrosus, and cartilage endplate. The gelatinous nucleus pulposus is in the center of the disc, and its secreted extracellular matrix is mainly composed of aggrecan and collagen type II. It can effectively maintain the osmotic pressure within the disc as well as the normal height of the disc. Annulus fibrosus which encircled nucleus pulposus is a ring of elastic fibers and fibrocartilage. Cartilage endplate is a thin layer of hyaline cartilage, separating the vertebrae and intervertebral disc and preventing the prolapse of nucleus pulposus from intruding into the adjacent vertebral body.^[4–6]^

Currently, the main treatments for DDD include surgery, conservative therapy, and biological therapy.^[7]^ Surgical treatment aims to relieve pain syndrome by removing herniated intervertebral disc tissue compressing nerve root, but it may cause loss of intervertebral disc height or recurrence of intervertebral disc prolapse; therefore, this treatment does not fundamentally cure or restore the physiological function of intervertebral disc.^[8]^ Conversely, biological treatment with cell therapy in particular can retard or reverse intervertebral disc degeneration by injecting stem cells directly into the disc, thus maintaining proper disc function.^[9,10]^

It has been reported in recent literatures that stem cells can be extracted from nucleus pulposus, annulus fibrosus, and cartilage endplate regions within the lumbar intervertebral disc, and these stem cells are equipped with different degrees of stem cell-genes expression of osteogenic, chondrogenic, and adipogenic, differentiation capacity.^[11]^ However, there was no literature studying the difference of biological characteristics of these stem cells (NPSCs, AFSCs, and CESCs) within the same degenerated human intervertebral disc. This study compares biological characteristics of 3 kinds of stem cells and clarifies potentials of NPSCs, AFSCs, and CESCs in reversing the degeneration of intervertebral disc.

## 2 Materials and methods

### 2.1 The main materials and equipment in experiment

Cell counting kit-8 (CCK-8, Dojindo, Japan); DMEM-LG, DMEM-F12 medium (Gibco); human stem-cell osteogenic induced medium, human stem-cell chondrogenic induced medium, human stem-cell adipogenic induced medium (Gibco); Bio-Rad Real Time PCR System (Biometra).

### 2.2 Samplings

After approval of the ethics committee of first affiliated hospital of Soochow University and all patients signing informed documents, we collected 15 cases of lumbar intervertebral disc tissues removed in surgery (Table 1).

**Table 1:**
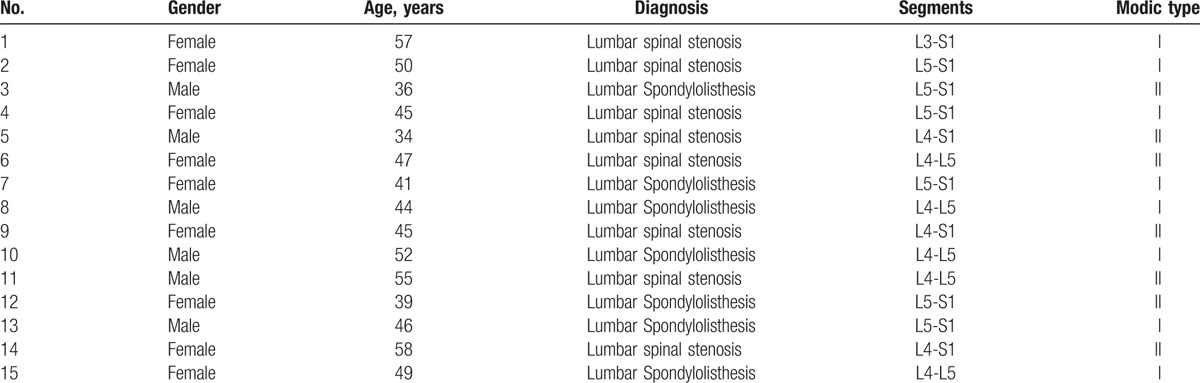
Source of tissue specimen.

### 2.3 Isolating and fostering CESCs, AFSCs, and NPSCs in degenerated intervertebral disc

Doctors with rich experience put the removed discs tissue into the centrifuge tube that contains DMEM-F12 medium, and then seal the tube and put it into the sample ice barrel. The centrifuge tube holding the sample was then placed on a sterile operating table and washed clean with PBS.

In a sterile environment, we removed the remaining ligament with eye scissors and cut nucleus pulposus, annulus fibrosus, and cartilage endplate tissues into fragments with size of 1 mm × 1 mm × 1 mm. The sample was then digested by 0.25% trypsin-EDTA in the 37 °C, water bath for 10 minutes, and then fostered in a containment with 0.2 mg/mL collagenase II and 0.1 mg/mL collagenase I in a humidified incubator at 37 °C, 5%CO_2_ for 2 hours. The suspension was centrifuged at 1000 rpm for 5 minutes. Three kinds of cells filtered by a 200 mesh filter was placed in 6-well plates separately and maintained in DMEM-LG supplemented with 10% FBS, 100 U/mL penicillin, and 100 mg/mL streptomycin. First medium was changed after 5 days, after which the medium was changed 2 times per week. When the primary cells reached the 85% to 90% of plate, they are harvested using 0.05% trypsin-EDTA and passaged with 1:3.

### 2.4 Observation of cell morphology

When 80% confluence of primary cells (P0) was achieved under observation, a photograph was made by the inverted microscope. After purification, the third passage cells were also photographed and recorded.

### 2.5 Curves of stem cell growth

We divided well-growing cells of the 2nd and 3rd generation into cell suspension with the concentration of 5 × 10^4^ cells/mL, and seeded into 96-well plates with 200 μL each hole. Then, we used the CCK-8 assay to check optical density (OD) values at the 1th, 3rd, 5th, 7th, 9th, 11th, 13th, 15th day separately.

### 2.6 Adipogenic differentiation

We seeded 2 mL NPSCs, AFSCs, and CESCs-suspension at passage 3 or 4 into 6-well plates according to 2 × 10^4^ cells/mL, and placed them in a humidified incubator at 37 °C with 5% CO_2_. After 3 days, we replaced basic medium with adipogenic-induced medium and changed once per 3 days. We used oil red O staining for observation through 3-weeks induction.

### 2.7 Osteogenic differentiation

Two milliliters of NPSCs, AFSCs, and CESCs-suspension at passage 3 or 4 was seeded into 6-well plates according to 1 × 10^4^ cells/mL and placed in a humidified incubator at 37 °C with 5% CO_2_. When fused by 90%, we sucked the basic media out and joined the osteogenic-induced medium in. We changed it 2 times per week and repeated it for 3 weeks and colored cells by alizarin red S.

### 2.8 Chondrogenic differentiation

We seeded 2 mL NPSCs, AFSCs, and CESCs-suspension at passage 3 or 4 into 15 mL centrifuge tubes according to 1 × 10^6^ cells/mL. After centrifugation, we dropped 2 mL chondrogenic-induced medium into the tubes slowly. Then, we changed the medium 3 days a week and repeated it for 3 weeks and used toluidine blue coloring at last.

### 2.9 Comparison of the expression of different lineage differentiation-related genes

The difference on the expression of AFSCs, NPSCs, and CESCs about osteogenic, chondrogenic, and adipogenic differentiation were detected by RT-PCR. All procedures were carried out according to instructions of reagents. The total RNA was extracted with a standard solution of Trizol. If the OD260/280 value of RNA measured by UV spectrophotometry was more than 1.8, the RNA was changed into cDNA through reverse transcriptase and amplified. 2^−ΔΔ Ct^ was used to calculate the relative expression of osteogenic, chondrogenic, and adipogenic differentiation-related genes. All primer sequences were generated by Primer 5.0 software (Table 2).

**Table 2:**
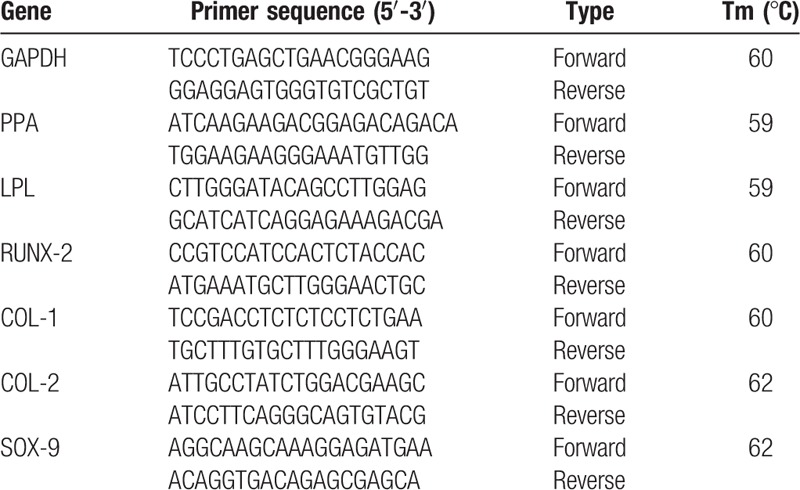
Primer sequence for RT-PCR detection.

## 3 Results

### 3.1 Morphological observation of NESCs, AFSCs, and CESCs

**Figure 1:**
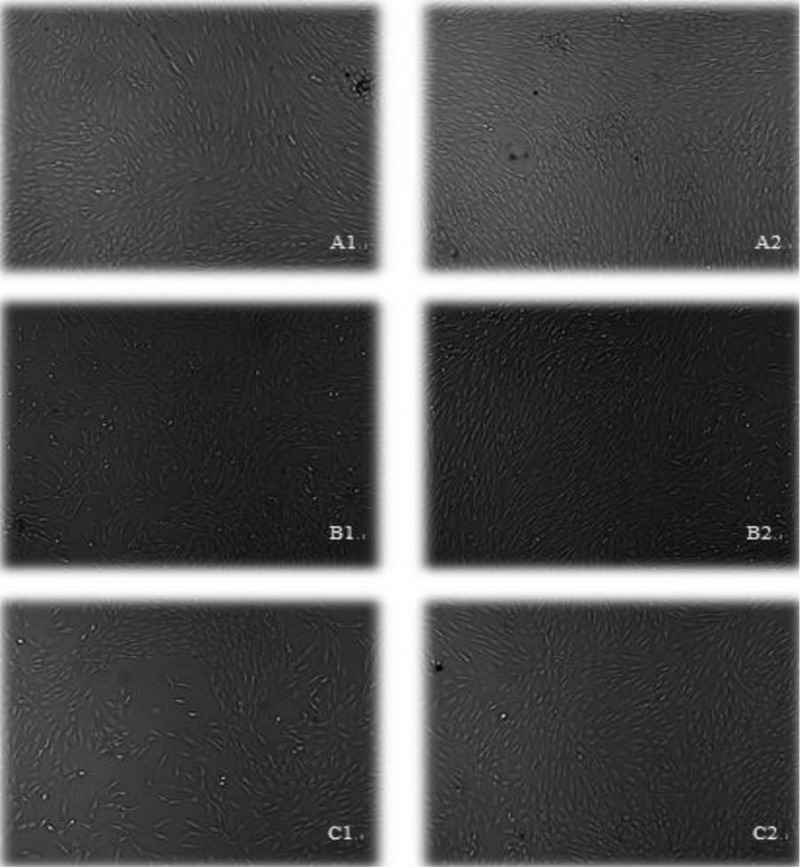
Amplification of 3 kinds of stem cells cultured in vitro 50×. A1: NPSCs at passage 0, A2: NPSCs at passage 3; B1: AFSCs at passage 0, B2: AFSCs at passage 3; C1: CESCs at passage 0, C2: CESCs at passage 3. AFSCs  =  annulus fibrosus-derived stem cells, CESCs  =  cartilage endplate-derived stem cells, NPSCs  =  nucleus pulposus-derived stem cells.

Primary cells of NESCs, AFSCs, and CESCs floated in the basic medium in round balls after initial inoculation. All cells grew around rings of the medium after 4 to 7 days and in short rod shape. Most of the primary cells were spindle-shaped, but not fully expanding. No significant difference was found in cell morphology. Fostered to 3rd generation, 3 kinds of stem cells derived from the disc appeared to be more uniform and more performance of fibroblast-like cells (Fig. 1).

### 3.2 Comparison of the growth curve of NPSCs, AFSCs, and CESCs

**Figure 2:**
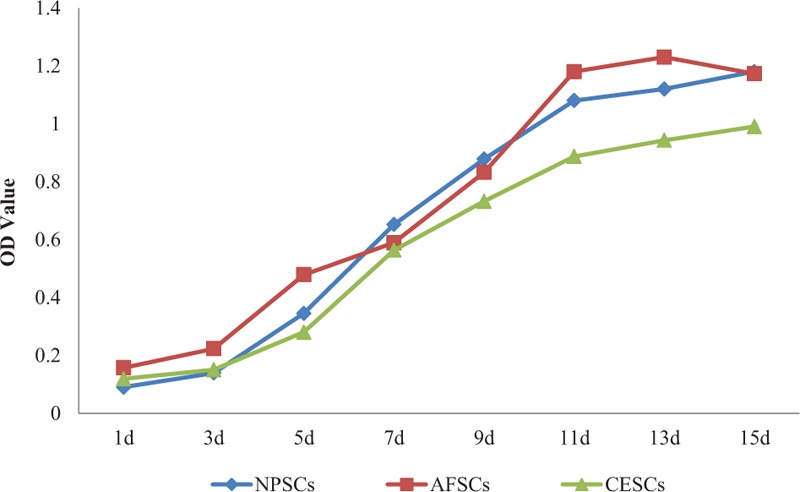
Comparison of the growth curves of NPSCs, AFSCs, and CESCs. AFSCs  =  annulus fibrosus-derived stem cells, CESCs  =  cartilage endplate-derived stem cells, NPSCs  =  nucleus pulposus-derived stem cells.

The CCK-8 assay was used to detect the proliferative rate of 3rd generation cells, and the result showed that the incubation period of the 3 kinds of stem cells is about 3 days. After entering the logarithmic growth phase, the proliferating rates of NPSCs and AFSCs were similar, and slightly faster than that of CESCs. After 5 to 6 days of logarithmic growth, all NPSCs, AFSCs, and CESCs entered the phase of platform growth, and cell proliferation rates leveled off (Fig. 2).

### 3.3 Different lineage differentiation of NPSCs, AFSCs, and CESCs and the difference of related gene with RT-PCR

**Figure 3:**
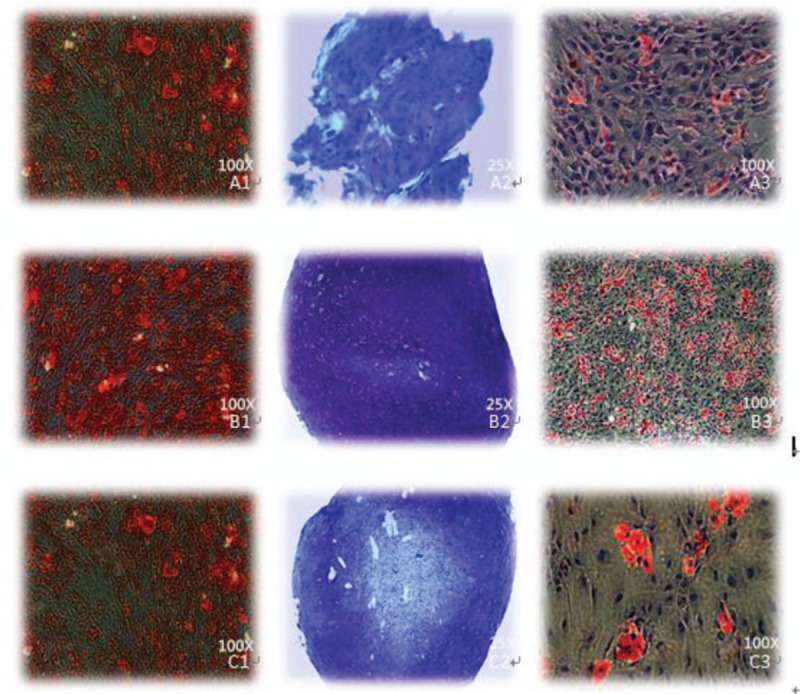
Different staining results of 3 kinds of stem cells in human intervertebral disc A1: alizarin red S staining of NPSCs, A2: toluidine blue staining of NPSCs, A3: oil red staining of NPSCs; B1: alizarin red S staining of AFSCs, B2: toluidine blue staining of AFSCs, B3: oil red staining of AFSCs; C1: alizarin red S staining of CESCs, C2: toluidine blue staining of CESCs, C3: oil red staining of CESCs. AFSCs  =  annulus fibrosus-derived stem cells, CESCs  =  cartilage endplate-derived stem cells, NPSCs  =  nucleus pulposus-derived stem cells.

**Figure 4:**
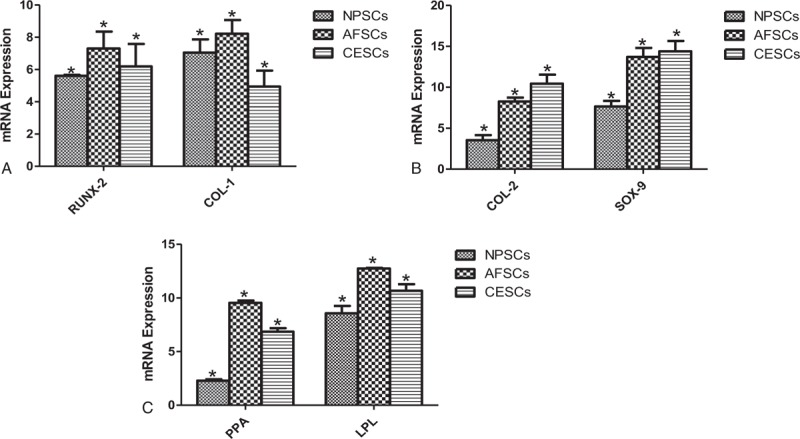
(A) The RT-PCR result of NPSCs, AFSCs, and CESCs after osteogenic differentiation. (B) The RT-PCR result of NPSCs, AFSCs, and CESCs after chondrogenic differentiation. (C) The RT-PCR result of NPSCs, AFSCs, and CESCs after adipogentic differentiation. AFSCs  =  annulus fibrosus-derived stem cells, CESCs  =  cartilage endplate-derived stem cells, NPSCs  =  nucleus pulposus-derived stem cells, RT-PCR  =  real-time polymerase chain reaction.

Extracellular matrix and rock salt of NPSCs, AFSCs, and CESCs gradually deposited after osteogenic differentiation, formed a plurality of black granular calcium nodules. Alizarin red S staining result for 21-days osteogenic induction indicated all stem cells were stained red (Fig. 3). Then, the differentiation outcomes of NPSCs, AFSCs, and CESCs in COL-1 and RUNX-2 genes were compared, and proved that NPSCs were significantly worse than AFSCs and CESCs in the RUNX-2 gene (Fig. 4A).

NPSCs, AFSCs, and CESCs were stained blue with toluidine blue after chondrogenic induction for 21 days, and the staining effect was slightly different (Fig. 3). The chondrogenic differentiate of 3 stem cells in COL-2 and SOX-9 genes existed difference. The expression of CESCs in these 2 genes was stronger than that of the other 2 groups, followed by AFSCs and NPSCs (Fig. 4B).

After 21-days’ adipogenic induction of NPSCs, AFSCs, and CESCs, oil red O staining results showed that 3 kinds of stem cells were all stained red and the staining effect of AFSCs was stronger than that of CESCs and AFSCs (Fig. 3). The adipogenic differentiational genes (PPA, LPL) of 3 kinds of stem cells were analyzed respectively, and the results showed that the expression of the 2 genes by AFSCs was slightly stronger than the other two cells. And the expression of the LPL gene by CESCs was the second highest and that of NPSCs was slightly worse (Fig. 4C).

## 4 Discussion

Nowadays, with the change in people's living environment and working styles, learning, entertainment, and travel make people sit for relatively long time and exercise for relatively short time, causing common clinical diseases of lumbar muscle strain and intervertebral disc herniation. It does not only disrupts patients’ life and work, but also increases the mental and economic burden, being the problems mostly urgent to be solved.^[12,13]^ At present, the most commonly clinical treatments of lumbar disc herniation are conservative treatment and surgical treatment, such as bed rest, traction, discectomy, and so on. These treatments can only temporarily relieve symptoms, but not stop or reverse the further degeneration of the intervertebral disc.^[14,15]^

Recent studies have found that stem cells have the ability of self-replication and multidirection differentiation, and are more active than the ordinary cells in immunogenicity or the secretion of extracellular matrix. So stem cell therapy has gradually become one of the most promising solutions of tissue engineering and regenerative medicine research.^[16–18]^

Stem cells were found in many tissues of the human body (bone marrow, muscle, adipose tissue).^[19]^ With the development of stem cell research, some adult tissues that were previously considered to be poor in repair capacity were found to have stem cells: nerve tissue, myocardial tissue, and so on.^[20,21]^ At present, the bone marrow mesenchymal stem cells are the seed cells for more research, which have the ability to differentiate into adipocytes, osteoblasts, and chondrocytes in vitro.^[22,23]^ However, it often brings patients and physicians unnecessary trouble of research stagnating because of the difficulty in obtaining and limited sources. A large number of disc specimens taken out of the interbody fusion have given people a new way of thinking: does the intervertebral disc also exist in stem cells of self-repair and proliferation ability? If yes, it does not require additional surgical procedures and avoids immune rejection from heterologous cells. Risbud et al^[24]^ first discovered a class of cells derived from the degenerated intervertebral disc that can express the surface antigen molecules of stem cells. Brisby et al^[25]^ used immunohistochemical methods and proved that the cells were NPSCs, which can positively express CD73, CD90, CD105 and negatively express CD45, C34, HLA-DR. The cells could be induced into adipocytes, osteoblasts, and chondrocytes, which provide a new method for the cell transplantation of degenerated intervertebral disc. Then, AFSCs and CESCs were successfully separated. It demonstrates that they have multiple differentiation potentials and the expression of cell surface markers with the same as other stem cells.^[26–29]^

Based on the sensitivity of MRI to the disc signal change, the specific indicators Modic syndrome (I-III type) could reflect changes of the intervertebral disc degeneration. Modic I and II suggest that there is inflammation and edema in the bone marrow; Modic III suggests that there may be a possibility of Subchondral osteosclerosis.^[30,31]^ In this experiment, we collected the specimen into the inclusion for the age of less 60 years old with Modic I or II-type patients for stronger capacity of cell proliferation and regeneration.

We use a new enzyme digestion method to digest the intervertebral disc tissue in our experiment. First, the sample was digested with 0.25% trypsin-EDTA in 37°C water bath for 10 minutes, then with 0.2 mg/mL collagenase II and 0.1 mg/mL collagenase I in a humidified incubator at 37 °C, 5%CO_2_ for 2 hours. Compared with other methods, this method has the advantages of simple operation, consuming short time and strong proliferation rate. The cell activity of NPSCs, AFSCs, and CESCs was identified by the CCK-8 assay at passage 2–3. Three kinds of stem cells grew by the “S” curve, which showed NPSCs and AFSCs were more active than CESCs. We wondered that the difference of activity between the 3 kinds of stem cells may be relevant to the difficulty to digest cartilage endplate and to obtain the cartilage endplate-derived stem cells.

Subsequently, we have the different lineage differentiation of 3 kinds of stem cells and expression detection of the related genes. The results showed that NPSCs, AFSCs, and CESCs could be induced successfully into adipocytes, osteoblasts, and chondrocytes. Then, we used different staining methods to compare the differentiation effect of 3 kinds of stem cells. It is proved that the AFSCs have more significant effects. The result of RT-PCR is also consistent with this argument. We conclude that it may be associated with a greater proportion of the annulus fibrosus in the intervertebral disc, and less influence on the degeneration of the intervertebral disc, thereby more likely to maintain the stem cell phenotype, and more expression of the stem cell.
